# Supplementary material for: The low complexity linker of DNAJB6b is key to its anti-amyloid function
Source: QRB Discov. 2025 Dec 2;6:e25. doi: 10.1017/qrd.2025.10016 (PMC12722041; doi:10.1017/qrd.2025.10016)
Supplement: Merkelis et al. supplementary material [file S2633289225100161sup001.docx]

**The low complexity linker of DNAJB6b is key to its anti-amyloid function**

Timas Merkelis^12^, Ulf Olsson^1^, Sara Linse^2^.

^1^Physical Chemistry, Lund University, Sweden

^2^Biochemistry and Structural Biology, Lund University, Sweden

**Supplementary information.**

**S1.** **Expression of the EDDIE-linker fusion construct**

The 110-residue linker of human DNAJB6b was cloned in Pet3a vector in fusion with the self-cleavable nPro mutant, EDDIE [1], with *Escherichia coli* (*E. coli*)- optimised codons. Gene synthesis and cloning was purchased from Genscript, Piscataway, New Jersey).

The plasmid was transformed into Ca^2+^-competent *E. coli* BL21 DE3 pLysS star cells using heat shock and spread on LB agar plates with 50 mg/L ampicillin and 30 mg/L chloramphenicol. Single colonies were picked for 50 mL day cultures in LB medium with 50 mg/L ampicillin and 30 mg/L chloramphenicol culture in 250 mL baffled flask. After 8 h, the equivalent of 0.5 mL at OD600 = 0.8 was transferred from each day culture to a separate 500 mL overnight autoinduction medium (prepared as described [2]) with 50 mg/L ampicillin and 30 mg/L chloramphenicol culture in 2.5 L baffled flasks, and grown for 15 h at 37oC with 125 rpm orbital shaking. The cultures were harvested by centrifugation at 6000 rpm in a JLA 8.1000 rotor and the pellet stored frozen at -80°C.

The expression of the gene yields the following protein sequence, with the DNAJB6b linker (to be obtained after cleavage from EDDIE) in blue:

MELNHFELLYKTSKQKPVGVEEPVYDTAGRPLFGNPSEVHPQSTLKLPHDRGEDDIETTLRDLPRKGDCRSGNHLGPVSGIYIKPGPVYYQDYTGPVYHRAPLEFFDETQFEETTKRIGRVTGSDGKLYHIYVEVDGEILLKQAKRGTPRTLKWTRNTTNCPLWVTSC**GGGGGGSHFDSPFEFGFTFRNPDDVFREFFGGRDPFSFDFFEDPFEDFFGNRRGPRGSRSRGTGSFFSAFSGFPSFGSGFSSFDTGFTSFGSLGHGGLTSFSSTSFGGSG**

**S2. Purification of the linker fragment**

**Sonication:** Cells from 1 L ON culture were sonicated 4 times in 80 mL 10 mM Tris/HCl, DNase, pH 8.0 for 2 minutes (50% duty cycle at 1 s on, 1 s off) and centrifuged after each sonication for 7 min at 15000 rpm in JA 25.50 rotor. Using a spatula, brown matter was scraped off from the top of the pellet after third and fourth sonication and discarded.

**Ion exchange 1 (iex1):** The remaining light grey pellet after the fourth sonication and centrifugation was dissolved in 100 mL 8 M urea, 20 mM Tris/HCl, 2 mM EDTA, 10 mM DTT, pH 8.0. The solution was diluted with 100 mL water and pumped onto a 20 mL DEAF FF column equilibrated in 4 M urea,10 mM Tris/HCl, 1 mM EDTA, 1 mM DTT, pH 8.0 (buffer A) using an FPLC system (BioRad DuoFlow) with on-line conductance measurement and quadrature absorbance detection. The column was eluted using a linear NaCl gradient in buffer A from 0-0.135 M NaCl over 150 mL, and then isocratic elution at 0.135 M NaCl for 150 mL followed by a final gradient from 0.135– 0.5 M NaCl over 100 mL. The elution was monitored by recording the conductivity as well as the absorbance at 214, 260 and 280 nm. Fractions of 5 mL were collected and analyzed by SDS PAGE (using pre-cast Novex Tris/Tricine 10-20 % polyacrylamide gels). Fractions dominated by the expressed fusion protein were pooled.

**Cleavage:** The pooled fractions from iex1, total volume 54 mL, were diluted with the same volume of 1 M Tris/HCl, 1 mM EDTA, 5 mM DTT, pH 7.8 and dialyzed against 1 M Tris/HCl, 1 mM EDTA, 5 mM DTT, pH 7.8 at 4°C in 3 shifts for 66 h using dialysis tubing with 3500 Da cutoff (boiled 4 times in distilled water before use). The tubing was opened and the solution was centrifuged for 10 minutes at 9000 rpm in 50 mL Falcon tubes in a BioFuge centrifuge at 4°C. The supernatant mainly contained cleaved EDDIE and was discarded. The pellet was washed in 10 mM Tris/HCl, 1 mM EDTA, pH 8.0, centrifuged again, second supernatant discarded, and the pellet brought forward to the next step.

**Ion exchange 2 (iex2):** The pellet after cleavage was dissolved in 50 mL 8 M urea, 10 mM Tris/HCl, 1 mM EDTA, pH 8.0, and diluted with 50 mL H_2_O, passed through a CM FF column and pumped onto 5 mL QHP column, both columns equilibrated in 4 M urea, 10 mM Tris/HCl, 1 mM EDTA, pH 8.0 (buffer B), using the FPLC system. The CM FF column was removed and the QHP column eluted using a linear NaCl gradient in buffer B from 0-0.1 M NaCl over 200 mL. The elution was monitored by recording the absorbance at 214, 260 and 280 nm, and 4 mL factions were collected and analyzed by SDS PAGE. Fraction dominated the linker were pooled.

**Hydrophobic interaction chromatography (HIC):** The pooled fractions from iex2, were supplemented with 0.3 M ammonium sulphate (AMS) and loaded in portions onto a 5 mL Phenyl Sepharose HP column equilibrated in 2 M urea, 10 mM Tris/HCl, 1 mM EDTA, pH 8.0 (buffer C) with 0.3 M AMS using a hand syringe. The sample was washed with 0.3 M AMS in buffer C and eluted with eluted with buffer C. The elution was monitored by by SDS PAGE. Fraction dominated the linker were pooled.

**Size exclusion chromatography:** Samples after HIC were loaded onto a 26/600 mm Superdex 75 column operated in 2 M urea, 20 mM sodium phosphate, 0.2 mM EDTA, pH 8.0. The elution was monitored by recording the conductance and the absorbance at 214, 260 and 280 nm, and 2 mL factions were collected and analyzed by SDS PAGE and MALDI mass spectrometry. The peak fractions containing the linker were pooled, aliquoted, flash frozen and stored at -20°C.

**Obtained product:** The above procedures resulted in the following product:

GGGGGGSHFDSPFEFGFTFRNPDDVFREFFGGRDPFSFDFFEDPFEDFFGNRRGPRGSRSRGTGSFFSAFSGFPSFGSGFSSFDTGFTSFGSLGHGGLTSFSSTSFGGSG


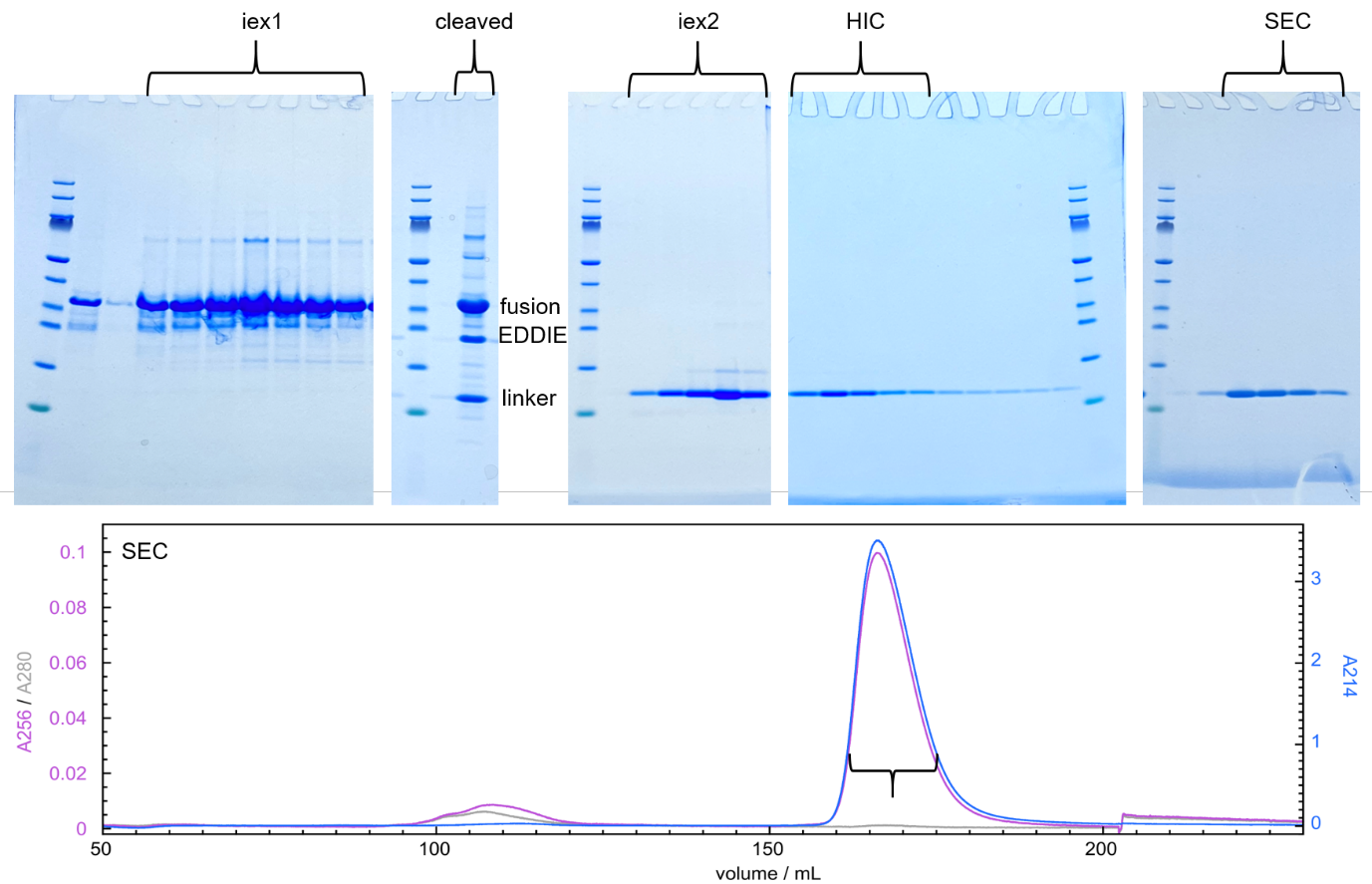


**Figure S1. Purification of the linker. Top row:** From left to right are shown SDS PAGE of the eluate from the first ion exchange of the fusion protein (iex1), after cleavage and separation of most of EDDIE by centrifugation (cleaved), after ion exchange of the cleaved product (iex2), after hydrophobic interaction chromatography (HIC) and after size exclusion chromatography (SEC). Collected fractions are indicated by curly brackets. **Bottom:** The chromatogram from the SEC run on Superdex75 26/600 with the absorbance at 214, 256 and 280 nm in blue, purple and grey, respectively. The lack of absorbance of the collected fractions is due to the lack of Tyr and Trp in the linker.

**Figure S2**. Dynamic light scattering results of 25 µM DNAJB6 linker measurements represented by decay rate Γ dependency on q^2^ with the corresponding linear fit, the slope of which represents the average diffusion coefficient of the measured particles. The sample is 2 days old, (incubated at room temperature after dialysis) with most of the sample being monomeric and aggregates being present. Plugging the diffusion coefficient into the Stokes-Einstein (1) equation yields an average hydrodynamic radius of 260nm. Pearson’s R for the linear fit y=ax – 0.99876.

$D=\frac{k_{B}T}{6\pi\eta R_{h}}$ (1)

Where D is the Diffusion coefficient of the particle in question, k_B_ is the Boltzmann constant, T is the absolute temperature, η is the solvent viscosity and R_h_ is the particle hydrodynamic radius.

**Figure S3**. FTIR of the linker aggregates after 2 weeks of incubation at room temperature. Analysis of the second derivative according to H. Yang et. al. 2015 yields: 64.6% β sheet (35.47% sheet, 29.15% turn), 21.4% helix (11.8% 3-10 helix, 9.8% α helix), 14% random coil.

**Figure S4**. Data from multiple CD measurements of the JB6 linker at different incubation times and concentrations. Although the amplitude of the spectrum changes, the shape remains the same.

**Figure S5**. HPLC aggregation data from a second JB6 linker batch. Measurements after 2 weeks were not possible due to degradation of the peptide.

 **Figure S6**. A repeat of the ThT fluorescence linker activity measurements against fibrillation. The linker was diluted into phosphate buffer to 200 nM with a final concentration of 100 nM after the addition of Aβ42. As the linker forms initial aggregates slowly on the time scale of 1 h, the activity varies greatly, with some wells in earlier times approaching the activity of the full protein and others approaching the previous results where the time before the addition of Aβ42 was not controlled.

 **Figure S7**. All curves for the time-controlled dilution of the JB6 linker experiment. As the linker aggregates it appears to lose activity, and the variance of the measurements drops significantly. Aβ42 aggregation without the addition of linker is represented in red.

**Figure S8**. The final fluorescence values for the time-controlled dilution of the JB6 linker experiment. As the linker aggregates and loses activity, the ThT fluorescence intensity increases, suggesting that the linker decreases the amount of ThT positive aggregates formed in the experiment and not just the aggregation rate

**Figure S9**. A ThT fluorescence experiment with α-synuclein with the presence of 4 µM JB6 linker (black) and without the addition of linker (red). The different panels indicate α-synuclein concentration. A-100 µM, B-70 µM, C-40 µM. The experiment included shaking the plate at 200rpm

**Figure S10**. Activity of the DNAJB6 linker equilibrated for 4 weeks on Aβ42 fibril formation evaluation using Thioflavin T fluorescence measurements. The concentration of Aβ42 is 4µM. Since the linker is aggregated in this state and the monomer concentration is negligible- there

**Figure S11**. ThT fluorescence over 170 hours with 400 nM or 14 µM JB6 linker and no amyloid. At the high concentration, ThT positive aggregates exist, but the intensity does not approach that of Aβ42 aggregates at 4 µM. An onset of aggregation can be seen at 400nM after 160 hours, which is outside the time scale of other kinetics experiments that were done.

**Figure S12.** A ThT titration experiment. The plot displays ThT fluorescence at 480 nm for JB6 linker samples at 17 µM were incubated for 1-4 weeks as specified in the legend. The samples were then diluted to 10 µM prior to the measurement. An excitation wavelength of 450 nm was used, and emission wavelengths from 475 to 500 were measured.

**Figure S13.** Amino acid composition comparison between the linkers of JB6 and JB8. Linker indicates the entire linker region while G/F and S/T indicate the corresponding regions of the linker.

**References**

**Linse S** (2020) Expression and Purification of Intrinsically Disordered Aβ Peptide and Setup of Reproducible Aggregation Kinetics Experiment. *Methods Molecular Biology* **2141**, 731–754.

**Wellhoefer M, Sprinzl W, Hahn R and Jungbauer A** (2013) Autoprotease Npro: Analysis of self-cleaving fusion protein. *Journal of Chromatography A* **1304**, 92–100.

**Yang H, Yang S, Kong J, Dong A and Yu S** (2015) Obtaining information about protein secondary structures in aqueous solution using Fourier transform IR spectroscopy. *Nature Protocols* **10**(3), 382–396.
